# IgA Nephropathy With Membranoproliferative Pattern and Resistance to Immunosuppressive Therapy in Two Patients With Cofactor I Pathogenic Variant

**DOI:** 10.1111/nep.70092

**Published:** 2025-07-11

**Authors:** Tommaso Mazzierli, Pamela Gallo, Costanza Giuliani, Elisabetta Pelo, Pietro Dattolo, Chiara Somma

**Affiliations:** ^1^ Department of Nephrology and Dialysis Santa Maria Annunziata Hospital Florence Italy; ^2^ Department of Biomedical Experimental and Clinical Sciences “Mario Serio” University of Florence Florence Italy; ^3^ Diagnostica Genetica Careggi University Hospital Florence Italy

**Keywords:** cofactor I, complement, genetic, IgA nephropathy

## Abstract

Complement system (CS) overactivation is one of the main causes of kidney damage in IgA nephropathy (IgAN), and it mainly involves the alternative pathway (AP). Additionally, pathogenic complement variants in CS‐related genes are reported in IgAN with associated thrombotic microangiopathy (TMA). Here we report two patients with IgAN presenting membranoproliferative pattern, isolated C3 hypocomplementemia, resistance to multiple lines of immunosuppressive therapy, familiarity for proteinuric chronic kidney disease and pathogenic rare variants in cofactor I (CFI). To the best of our knowledge, no other cases of IgAN patients with a similar phenotype and genotype were previously reported in the literature. This work highlights the essential role of deep phenotyping and genotyping in providing tailored treatment strategies in IgAN patients.

## 1 Introduction

Iga Nephropathy (IgAN) represents the most diagnosed primary glomerulopathy worldwide in patients undergoing kidney biopsy [1, 2].

Complement system (CS) is one of the main determinants in glomerular inflammation. However, it is unclear how IgA‐immune complexes can activate complement pathways. Several studies show complement overactivation in IgAN patients [3, 4] and link it to a rapid progression of kidney disease and a bad outcome [5, 6]. The key role of the CS was supported by evidence of the effectiveness of anti‐complement therapy in the treatment of IgAN [7]. In the literature, patients with IgAN and pathogenic variants in complement genes have been reported, mainly involving Cofactor and Cofactor H (CFH) related protein 5 (CFHR5) [8, 9, 10].

In this paper, we present two cases of IgAN that share similar histological features in kidney biopsy (e.g., the presence of a membranoproliferative lesion pattern), isolated C3 hypocomplementemia, resistance to immunosuppressive therapy, familial renal disease and positive exome sequencing for pathogenic variants in complement Cofactor I (CFI).

## 2 Case 1

A 25‐year‐old male was admitted to our Nephrology Department in October 1997 for the incidental finding of severe hypertension, subnephrotic proteinuria (2.8 g/24 day), microhaematuria, and acute kidney injury (sCr 2 mg/dL, basal value 3 months before 0.8 mg/dL). Autoimmune and infectious disease screening were negative with isolated C3 hypocomplementaemia (60–65 mg/dL, n.v. > 90 mg/dL). No signs of haemolytic anaemia were present at the onset. Thereafter, a kidney biopsy was performed. The histopathologic examination showed 19 glomeruli with diffuse mesangial (M1) and endocapillary (E1) hypercellularity, with two fibrocellular crescents. No glomerular sclerosis (S0) and a moderate chronic tubulo‐interstitial damage (30%‐T1) were detected. The arterioles showed increased thickness with rare lumen thrombi. The immunofluorescence (IF) showed a strong mesangial staining for IgA (3+) with codominant C3 (3+). No significant glomerular staining for IgG, IgM and C1q was detected. Electron microscopy was not performed. The patient was diagnosed with IgAN and treated with pulse methylprednisolone, oral corticosteroids and intravenous cyclophosphamide. Despite the treatment, he developed a worsening of the proteinuria and a rapid progression to end‐stage renal disease. He started haemodialysis in 1999. In 2005, he received a living kidney donor transplant from his healthy sister, but he had an initial acute cell‐mediated graft rejection treated with steroids and thymoglobulin. He partially recovered renal function, which remained stable during the following 6 years (sCr 1.6–1.9 mg/dL and proteinuria 0.8–1 g/day). In 2012, renal function worsened again (sCr 3.5–4 mg/dL) and proteinuria reached nephrotic range (5–6 g/day) and a second graft biopsy was performed; it showed diffuse proliferation of mesangial cells with slight lobular accentuation, capillary wall thickening, and focal intracapillary hypercellularity. There was also arteriolar hyalinosis. The IF showed moderate codominant IgA and C3 (2+) mesangial and parietal staining. C4d deposition and negative donor‐specific antigen were reported, excluding antibody‐mediated rejection. Electron microscopy was not performed. These findings have been interpreted as IgAN recurrence and treated with steroids and multiple lines of immunosuppressive therapy, cyclophosphamide and calcineurin inhibitor) without achieving improvement in renal function.

**FIGURE 1:**
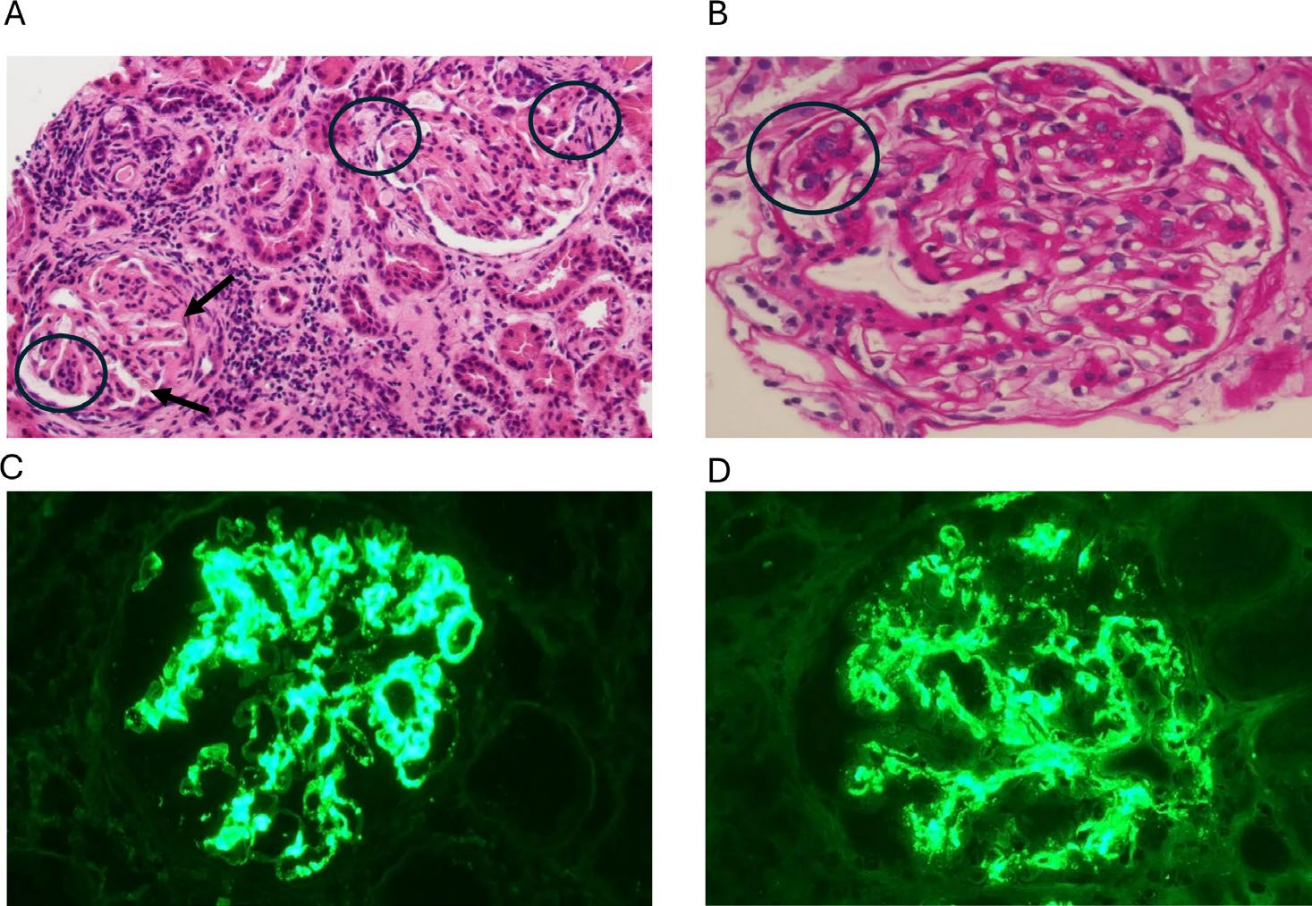
(A and B) Diffuse proliferation of mesangial cells with slight lobular accentuation (arrows) with focal endocapillary hypercellularity (circles); (C and D) Positive dominant mesangial and parietal IgA and C3 staining.

In 2017, considering the lack of response to therapies, a third graft biopsy was performed. The histological pattern was similar to the previous biopsies (Figure 1A,B), but a change in IF was observed with codominant strong IgA and C3 (3+), moderate IgG and IgM (2+) staining in mesangial, subendothelial and parietal areas (Figure 1C,D). Electron microscopy showed the presence of mesangial and subendothelial deposits with glomerular basement membrane (GBM) thickening. The patients at the time of third biopsy present with decreased C3 level (65–70 mg/dL), normal C4 values (20–25 mg/dL, n.v. > 15 mg/dL) and an increased complement deposition on activated endothelial cells (254%, v.n < 150%). The patient progressed to ESRD in 2022 and started peritoneal dialysis (PD).

Furthermore, after kidney donation, the patient's sister developed severe hypertension, proteinuria, and hematuria and subsequent chronic kidney disease. She also presented persistent isolated C3 hypocomplementemia (70–75 mg/dL). She also had increased complement deposition on activated endothelial cells (213%, v.n < 150%). Considering the solitary kidney, we decided not to perform a kidney biopsy. She is currently treated with maximal nephroprotective therapy.

Taking into account the familiarity for chronic kidney disease, a genetic test (whole exome sequencing) was performed in the patient and his sister. Two pathogenic variants were detected in CFI [c.292A > G, p.(Thr98Ala) and c.490G > A, p.(Asp164Asn)] [11], one inherited from the mother and one from the father, both asymptomatic carriers.

## 3 Case 2

A 43‐year‐old female with a history of IgAN onset in childhood (13 years) was referred to our Nephrology Department to start peritoneal dialysis due to ESRD in 2013. In 1981, she presented nephrotic range proteinuria, macrohaematuria, and severe hypertension. No other clinical and biochemical parameters were available from the first hospital admission except for the biopsy report that showed the presence of mesangial proliferation (M1) in 14 glomeruli with mild interstitial fibrosis and tubular atrophy (T1, 20%). No endocapillary hypercellularity or glomerular sclerosis was detected (E0, S0). Vascular lesions included arterial fibrotic intimal thickening and arteriolar hyalinosis. The IF showed a codominant IgA and C3 positivity (2+) in mesangial areas. She underwent multiple lines of immunosuppressive treatment (pulse IV steroids, cyclophosphamide, mycophenolate mofetil and calcineurin inhibitor) with no response, persistence of nephrotic range proteinuria, and progressive loss of kidney function. Considering the absence of response to immunosuppressive therapy, she was biopsied a second time in 1983. At that time, blood exams showed an isolated C3 hypocomplementaemia (70 mg/dL) while autoimmune and infectious disease screening (HBV, HCV and HIV) was negative. The second biopsy revealed 17 glomeruli (five of which globally sclerosed) with diffuse mesangial hypercellularity. Nine glomeruli showed segmental duplication of the GBM. Compared to the first biopsy, similar patterns were reported in vascular and interstitial areas. Vice versa, the IF showed a variable granular peripheral capillary wall and significant mesangial staining for IgA and C3 (3+) and a moderate staining for IgM and IgG (2+). There was no staining for C1q. Thereafter, she developed end stage kidney disease (ESKD) and started peritoneal dialysis in 2014.

In 2019, during the evaluation as a potential kidney donor, her brother was diagnosed with proteinuric nephropathy (0.4–0.5 g/24 h), hypertension, and chronic kidney disease (sCR 1.2 mg/dL, eGFR 72 mL/min/1.73 m^2^). He had a normal complement level and activity. He refused to undergo kidney biopsy. Considering the familiarity with kidney disease and the presence of IgAN with pathologic features of membranoproliferative glomerulonephritis, a clinical exome sequencing was performed in both patients. The novel rare c.1007G > A p.(Arg336Gln) variant was detected in CFI. Recently, Zhang et al. [12] described variants in CFI affecting the 336 amino acid residue. Authors postulate that these variants lead to a circulating unprocessed FI and should be classified as likely pathogenic. Unfortunately, parents were not available to complete an appropriate segregation analysis. Considering the positivity for the CFI variant, in vivo and in vitro complement activity were evaluated in the patient, showing decreased C3 level (60 mg/dL), increased complement deposition on activated endothelial cells (259%) and altered activity of the alternative pathway (AP) at Wieslab test (8%, n.v. > 10%).

## 4 Discussion

The role of CS in IgAN is well characterised with increased level and expression of complement protein (es. C3bi, C4d), impaired complement protein inhibitor function (es. CFH and CFHR5) and increased terminal complement product (C5b) in blood, urine and kidney tissue [13]. AP is the main pathway involved in the pathogenesis of IgAN [13].

In this case report, we describe two IgAN patients with similar genotypic and phenotypic features:

- Rare CFI pathogenic variant with associated altered AP activity that segregates with family variant segregation with kidney disease;
- Membranoproliferative pattern at kidney biopsy with co‐dominant IgA/C3;
- Resistance to standard immunosuppressive treatment.

The histological and complement‐related biochemical features are reported in Table 1.

**TABLE 1:** Main histopathological findings and complement abnormalities in Case 1–2 at last biopsy.

	Case 1	Case 2
Clinical features		
Complement factor 3 level, mg/dL, > 90 mg/dL	60–65 mg/dL	70 mg/dL
Complement factor 4 level, mg/dL, n.v. > 15 mg/dL	20–25 mg/dL	31 mg/dL
C5b9 endothelial deposition, 5, n.v. < 150%)	254%	259%
AH50, %, n.v. > 10%	n.a	8%
CH50, %, n.v. > 70%	n.a.	72%
MBL50, %, n.v. > 10%	n.a.	26%
Histological features		
Glomerular lesion pattern	Membranoproliferative pattern	Membranoproliferative pattern
Other reported lesions	Endoluminal thrombi (1°biopsy)	None
IF	IgA 3+	IgA 3+
	C3 3+	C3 3+
	IgG 2+	IgG 2+
	IgM 2+	IgM 2+
Genetic results	CFI (c.292A > G;p.Thr98Ala), (c.490G > A; p. Asp164Asn)	CFI c.1007G > A p.(Arg336Gln)

*Note:* AH50, CH50 and MBL50: Wieslab haemolytic assay.

Abbreviation: IF, immunofluorescence.

Patients with IgAN and pathogenic variants in complement genes have already been reported in the literature [13]. All the pathogenic variants are reported in CFH and CFHR5, which strictly regulate AP. Cases of patients with CFI pathogenic variants and glomerulopathies like thrombotic microangiopathy (TMA) and immune‐complex membranoproliferative glomerulonephritis have already been described in literature [14, 15]. Mauleman et al. [15] report that in those patients:

- The disease onset may be influenced and triggered by a combination of genetic, autoimmune, and environmental factors;
- The AP is the main altered complement pathway and the driver of kidney damage.

The IgAN pathogenesis is driven by the formation of IgA1‐containing immune complexes that are able to activate AP [2]. In our patients, the overactivation of the AP is suggested by a significant isolated C3 hypocomplementemia, the presence of a membranoproliferative pattern with co‐dominant C3 IF staining, and high in vivo or in vitro AP activity. We speculate that IgA1‐containing immune complexes act as the main driver for AP overactivation and, subsequently, the presence of altered CFI activity exacerbates this process, leading to sustained complement activation. This hypothesis is also supported in other research works [8, 16] where authors report patients with IgAN and rare pathogenic variants that present with development of TMA. As reported in literature [13], both glomerular injury patterns (membranoproliferative and TMA) share many characteristics such as the presence of extensive endothelial damage, massive complementary activation and can often coexist within the same biopsy.

The presence of CFI pathogenic variants could impact negatively on disease progression. In IgAN patients, biochemical and bioptical signs associated with the overactivation of the CS are associated with a worse renal outcome [5]. Our two cases, along with the other IgAN patients with pathogenic complement variants [8, 13, 17], exhibit a high prevalence of those risk factors indicative of complement overactivation, including them in the category of high risk of progression and unfavourable outcome.

Furthermore, our two patients demonstrated resistance to conventional immunosuppressive treatments. A similar resistance to traditional treatment protocols was described by Tortajada et al. [8] in five patients with rare complement variants and IgAN. Vice versa, three of them were treated with anti‐C5 therapy and showed a complete or partial recovery of kidney function. Similarly to these three patients, a resistance to immunosuppressive treatment is also reported in C3GN/IC‐MPGN with pathogenic complement variants [9, 15, 16].

Considering the recent availability in clinical practice of anti‐complement drugs approved for IgAN, the identification of patients harbouring complement gene pathogenic variants could become crucial in order to nominate them as first‐line candidates for treatment with those drugs.

**FIGURE 2:**
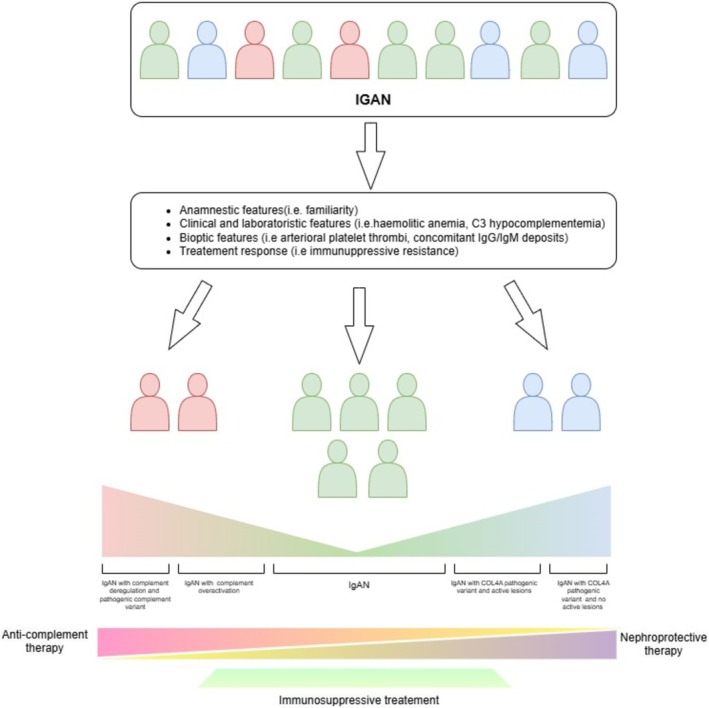
Proposal of a personalised therapeutic approach for IgAN patients based on clinical, biopsy and genetic features.

Finally, our two cases present both familiarity for proteinuric kidney disease and highlight the importance of considering an overlapping disease spectrum between IgAN and genetic kidney diseases, such as collagenopathies (Figure 2). In these cases, bioptical and genetic data should be integrated in order to define the best IgAN treatment and outcome.

In conclusion, our case report emphasises the significant role of the CS in the pathogenesis of IgAN. Both patients have rare pathogenic variants in the CFI gene, resulting in excessive activation of the alternative complement pathway. This may explain their lack of response to conventional immunosuppressive treatments. Our findings suggest that anti‐complement therapies could be a more effective approach for such patients. As suggested in literature [18], a deep phenotyping and genotyping (if necessary) are essential for providing tailored treatment strategies, potentially improving kidney function and long‐term prognosis.

## Ethics Statement

This article does not contain any study requiring ethical approval. Human‐related data used in this study were obtained from public databases in a totally anonymised and aggregated form. The results are appropriately placed in the context of prior and existing research. All authors have been personally and actively involved in substantial work leading to the paper.

## Conflicts of Interest

The authors declare no conflicts of interest.

## Data Availability

Research data are not shared.
